# Mapping the prevalence of severe acute malnutrition in Papua, Indonesia by using geostatistical models

**DOI:** 10.1186/s40795-022-00504-z

**Published:** 2022-02-14

**Authors:** Paul Jasper, Warren C. Jochem, Emma Lambert-Porter, Umer Naeem, Chigozie Edson Utazi

**Affiliations:** 1 Oxford Policy Management Limited, Level 3, Clarendon House, 52 Cornmarket Street, Oxford, OX1 3HJ UK; 2 School of Geography and Environmental Sciences, University of Southampton, Southampton, SO17 1BJ UK; 3 Southampton Statistical Sciences Research Institute, University of Southampton, Southampton, SO17 1BJ UK

**Keywords:** Severe acute malnutrition, Prevalence threshold, Bayesian geostatistics, Mapping, Papua, Indonesia

## Abstract

**Background:**

Severe acute malnutrition (SAM) is the most life-threatening form of malnutrition, and in 2019, approximately 14.3 million children under the age of 5 were considered to have SAM. The prevalence of child malnutrition is recorded through large-scale household surveys run at multi-year intervals. However, these surveys are expensive, yield estimates with high levels of aggregation, are run over large time intervals, and may show gaps in area coverage. Geospatial modelling approaches could address some of these challenges by combining geo-located survey data with geospatial data to produce mapped estimates that predict malnutrition risk in both surveyed and non-surveyed areas.

**Methods:**

A secondary analysis of cluster-level program evaluation data (*n* = 123 primary sampling units) was performed to map severe acute malnutrition (SAM) in Papuan children under 2 years (0–23 months) of age with a spatial resolution of 1 × 1 km in Papua, Indonesia. The approach used Bayesian geostatistical modelling techniques and publicly available geospatial data layers.

**Results:**

In Papua, Indonesia, SAM was predicted in geostatistical models by using six geospatial covariates related primarily to conditions of remoteness and inaccessibility. The predicted 1-km spatial resolution maps of SAM showed substantial spatial variation across the province. By combining the predicted rates of SAM with estimates of the population under 2 years of age, the prevalence of SAM in late 2018 was estimated to be around 15,000 children (95% CI 10,209–26,252). Further tests of the predicted levels suggested that in most areas of Papua, more than 5% of Papuan children under 2 years of age had SAM, while three districts likely had more than 15% of children with SAM.

**Conclusions:**

Eradication of hunger and malnutrition remains a key development goal, and more spatially detailed data can guide efficient intervention strategies. The application of additional household survey datasets in geostatistical models is one way to improve the monitoring and timely estimation of populations at risk of malnutrition. Importantly, geospatial mapping can yield insights for both surveyed and non-surveyed areas and can be applied in low-income country contexts where data is scarce and data collection is expensive or regions are inaccessible.

**Supplementary Information:**

The online version contains supplementary material available at 10.1186/s40795-022-00504-z.

## Background

Eradication of malnutrition is an important global challenge and remains central to the UN Sustainable Development Goals (UN SDGs): to end all forms of hunger and malnutrition by 2030 and meet internationally agreed targets on stunting and wasting in children under 5 years of age [1]. In 2019, it was estimated that approximately 6.9% of (or 47 million) children under the age of 5 years old were affected by wasting or acute malnutrition [1]. Approximately 14.3 million of these were estimated to be severely acutely malnourished in 2019 [2]. Severe acute malnutrition (SAM) is the most life-threatening form of malnutrition and is defined by a weight-for-height or length z-score (WHZ) < − 3 standard deviations (SD) below the World Health Organization (WHO) reference median (and/or a mid-upper arm circumference [MUAC] < 115 mm) [3]. Children with SAM are at immediate risk of death [4].

The prevalence of child malnutrition is generally established through large-scale household surveys that are run at multi-year intervals, including Demographic and Health Surveys (DHS), Multi-Indicator Cluster Surveys (MICS), Standardized Monitoring and Assessment of Relief and Transitions (SMART) surveys, and National Nutrition Surveys (NNS). In the context of low- and middle-income countries (LMICs), in particular, large-scale national surveys are associated with a number of limitations, including very high cost (the UN estimates that a single national survey costs at least $1 million per wave, with 2–4 survey waves required within a 10-year cycle to monitor indicators in line with the SDG agenda) [5]; high levels of aggregation (i.e. survey estimates are available at the national or first administrative level, as more granular estimates require larger samples at a higher cost); large time intervals between survey waves (national surveys are typically run at 2–5 year intervals); and gaps in area coverage in the estimates (due to security and access constraints).

Geospatial modelling approaches could address some of these challenges by combining geo-located survey data with geospatial data to produce mapped estimates that predict malnutrition risk in both surveyed and non-surveyed areas—at a higher resolution than what is directly possible with survey data. This approach has the potential to inform programming policy and decision-making by identifying priority areas (e.g. “hot spots” with higher risk). At present, no study has modeled SAM (separately from other malnutrition indicators) by using geospatial approaches, although, more broadly, a few studies have produced maps of malnutrition indicators. Two recent large-scale studies have exemplified the use of geospatial mapping: one mapped child growth failure (CGF) indicators (stunting, wasting and underweight) at high resolution (5 × 5 km) across districts in India from 2000 to 2017 [6], and the second study mapped CGF (at a 5 × 5 km resolution) on a broad scale across 105 LMICs from 2000 to 2017 [7]. Overall, the literature indicates heterogeneity of malnutrition risk at sub-national levels, and it suggests that finer-scale estimates could help identify areas that are of higher priority for CGF reduction.

In this study, we explored the use of geostatistical modelling techniques to produce high-resolution (1 × 1 km) prediction maps of SAM prevalence and the associated uncertainty estimates in Papua, the eastern-most province of Indonesia. Historically, Papua is a data-scarce environment, and data collection in the region is difficult owing to the insecurity from violent conflict and the geographical inaccessibility of some areas, which has been exemplified by the exclusion of Papua from some rounds of the Indonesian DHS survey series (e.g. in 2002/2003) [8]. Existing estimates of SAM prevalence in this province are not available at the district level. The 2018 Indonesian Basic Health Research (RISKESDAS) national household survey estimated that the prevalence of wasting in Papua was over 10% [9]. The baseline survey of the Child Grant (CG) program evaluation, implemented in late 2018, estimated that the overall prevalence of child wasting in the surveyed areas among Papuan children under 2 years of age was 20% [8]. In terms of health indicators, Papua is one of the poorest-performing regions in Indonesia [10].

For this study, georeferenced household survey data were analyzed in combination with geospatial data layers describing local contextual factors. We also explored the use of thresholds of SAM prevalence estimates in order to communicate the risk of SAM and to inform policy decisions in the region as they relate to SAM programming.

## Methods

**Fig. 1:**
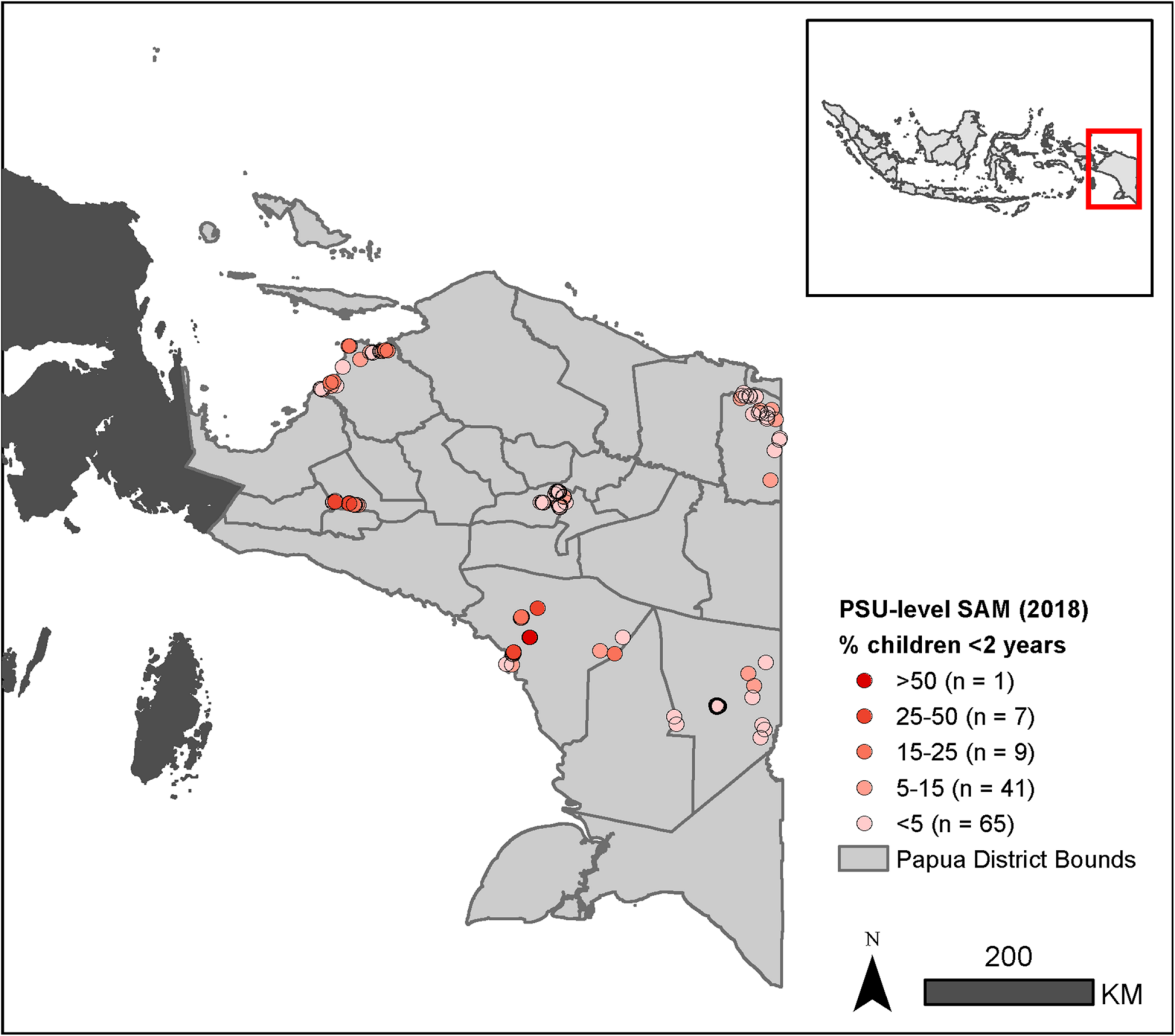
Percentage of surveyed children experiencing SAM in Papua, Indonesia within each PSU in 2018. Data shown are selected primary sampling unit (PSU) locations (*n* = 123) collected during the baseline survey of the BANGGA Papua evaluation.

The analysis in Papua (Fig. 1) used local data on malnutrition collected during the baseline survey of the UNICEF-funded evaluation of the Child Grant (CG) program, known as BANGGA Papua, conducted in late 2018 [8]. The study design for the BANGGA Papua evaluation initially selected six districts in Papua. A list of accessible and safe villages in each selected district served as the sampling frame for primary sampling units (PSUs), from which 20 PSUs were randomly selected per district. Within each village, a household listing exercise was conducted to identify eligible children and their households. Papuan children under the age of 2 years (0–23 months) with a Papuan caregiver were eligible for the study. These were households where the caregiver self-identified as an indigenous Papuan. A sample of 15 children was randomly selected in each PSU and the households were invited to join the study. The baseline survey included questions related to feeding and childcare, and anthropometrics were used to measure children’s weight and height, which were captured by enumerators specifically trained as anthropometric specialists. These measurements were also checked for quality (outliers in Z-scores and standard deviations), and the results indicated no quality concerns with the survey data.

We defined SAM in children as a weight-for-height or length z-score (WHZ) more than 3 standard deviations below the WHO reference median (3). A GPS location was also collected for each surveyed household, and for maps and spatial analyses, the centroid point location of the households in each PSU was used to represent the cluster of households. These “cluster” locations linked to each PSU are the unit of analysis in the geostatistical model. The data collection was approved by the Ethical Review Committee (ERC) at Oxford Policy Management Limited in August 2018. Secondary analysis of the data presented here was approved by the University of Southampton (ERGO II: 61645).

### Covariate data processing and selection

Prediction of SAM in locations without survey data draws on information from the spatial structure and patterns in SAM at the observation locations as well as from modelled relationships with covariate data. Many socioeconomic, demographic, environmental, and physical factors can directly or indirectly influence malnutrition and the spatial distribution and inequalities in SAM. In the statistical modelling framework, these ancillary factors act as covariates to help explain some of the observed spatial variation in SAM. When mapped as a geospatial layer, the covariates also aid in the prediction of SAM to non-surveyed areas. Therefore, in addition to being correlated with SAM, in order to be used as covariates in the statistical analysis, the data must be processed and mapped consistently for surveyed and non-surveyed locations across the entire study area.

We assembled a set of potential covariates based on a literature review of the factors that are associated with wasting and malnutrition and were mapped consistently throughout the study area (Supplementary Table 1, Additional File 1). The potential covariates were related to factors such as remoteness, measured as travel time to major settlements or the distance to travel infrastructure. Other factors included childhood diseases from modelled estimated risk of diarrhea [11] and malaria [12]. The geospatial data layers were reprojected and harmonized to a consistent 1 km × 1 km to support the predictions. A full list of the covariate layers and processing steps are reported in the supplementary materials (Additional File 1).

While many covariates could be plausibly included in the model, a parsimonious model using the fewest number of covariates is preferred to maximize predictive accuracy. The use of more covariates than is necessary introduces the risk of overfitting to the observed data, which yields worse predictions in non-surveyed areas. We implemented a multi-step process to reduce the number of covariates selected for our analysis. Covariate selection used non-spatial binomial generalized linear models (GLMs) to test the association with SAM. We also eliminated covariates that were highly correlated (⍴ > 0.8) by using Akaike Information Criterion (AIC). This type of pre-processing is commonly applied when building predictive geospatial models [13, 14]. Full details and results of covariate selection are presented in the supplementary materials (Additional File 1).

### Geostatistical model

The number of children experiencing SAM out of the total number of surveyed children at each cluster location was modelled using binomial spatial regression. Each survey cluster was associated with a selected set of covariates based on the GPS location of the cluster centroid. In addition to the covariates, which were treated as fixed effects, the model framework included a spatial random effect. The spatial effect is a zero-mean stationary Gaussian Process with a Matérn covariance function. This effect captures the tendency for cluster locations which are in close spatial proximity to have similar levels of SAM, a property referred to as spatial autocorrelation. As the distance between clusters increases, the spatial autocorrelation and the expected similarity between clusters decreases. The spatial effect serves several purposes in the model. First, the spatial autocorrelation in the data can arise from model misspecification via a shared risk factor that is not included in the covariate data. The spatial effect can account for this residual error by drawing on information in the patterns of the observed outcome locations. Second, the spatial effect smooths the predictions across the study area. In the absence of a strong relationship with the contextual covariates, the spatial effect creates a spatial interpolation among the clusters. The details of the model are given in Additional File 2.

The geostatistical model was estimated in a Bayesian framework using integrated nested Laplace approximations (INLA) for the latent Gaussian models [15]. The stochastic partial differential equation approach [16] was used for the spatial random effect to approximate a continuous spatial field. The use of Bayesian techniques allows quantification of the uncertainty in the model. To evaluate our models, we used a 10-fold cross-validation procedure. The model was refitted 10 times, each time omitting a different 10% of the survey clusters that were used as validation locations to compare the predictions. The coefficient of determination (R^2^) was used to evaluate predictive performance by comparing the relationship between observed and predicted values in the cross-validation as well as an in-sample comparison using the full set of observations.

The fitted model using all observations was used to predict the proportion of children experiencing SAM on a 1 × 1 km resolution grid from 1000 posterior samples. The gridded predictions include the mean of the posterior samples as the best estimate as well as the upper and lower 95% intervals of the predictions to express uncertainty around the prediction. The posterior samples for each grid cell were then aggregated to create district- and province-level estimates as the population-weighted mean of the grid cells falling within the areas (described in Additional File 2). The population data used for this step were modelled estimates of the total population and age-sex proportions calculated at a 1 × 1 km grid resolution [17]. Papua is ethnically diverse [18], and with no recent estimates of the indigenous Papuan population, we used the total population estimates to approximate this distribution.

We further examined the uncertainty in the predicted levels of SAM by calculating the probability that the levels of SAM in children under 2 years of age exceeded certain thresholds and mapping these levels across Papua. For these thresholds we used 2, 5, 10 and 15% cutoffs and applied them to the 1 km × 1 km gridded predictions of SAM. While no standard thresholds for SAM exist, the WHO considers wasting (WHZ < − 2 SD)—which includes both moderate acute malnutrition (MAM) and SAM—at or exceeding 15% to be critical from the standpoint of public health significance [19].

The model was implemented using R [20] and the R-INLA package [15] with the raster package [21] for spatial data handling.

## Results

On the basis of the survey observations collected in late 2018, a total of 107 out of 1508 surveyed children were identified as experiencing SAM (7.1%). Across 123 PSUs, the level of SAM in the surveyed children varied substantially from 0 to 76.9% (mean = 7.4%, standard deviation = 12.0). The variation in SAM between clusters showed geographic patterns that we explored with the geostatistical models (Fig. 1 and Supplementary Fig. 2, Additional File 1). The results of the covariate selection procedures identified annual precipitation, distance to conflicts, distance to settlements, distance to major roads, distance to urban areas, and travel time to urban centers as the best geographical predictors of SAM in Papua. We examined the residuals of a non-spatial binomial model using these covariates with a variogram (Supplementary Fig. 2, Additional File 1), and found some remaining spatial variation at shorter spatial ranges, suggesting the use of the geostatistical model.

**Fig. 2:**
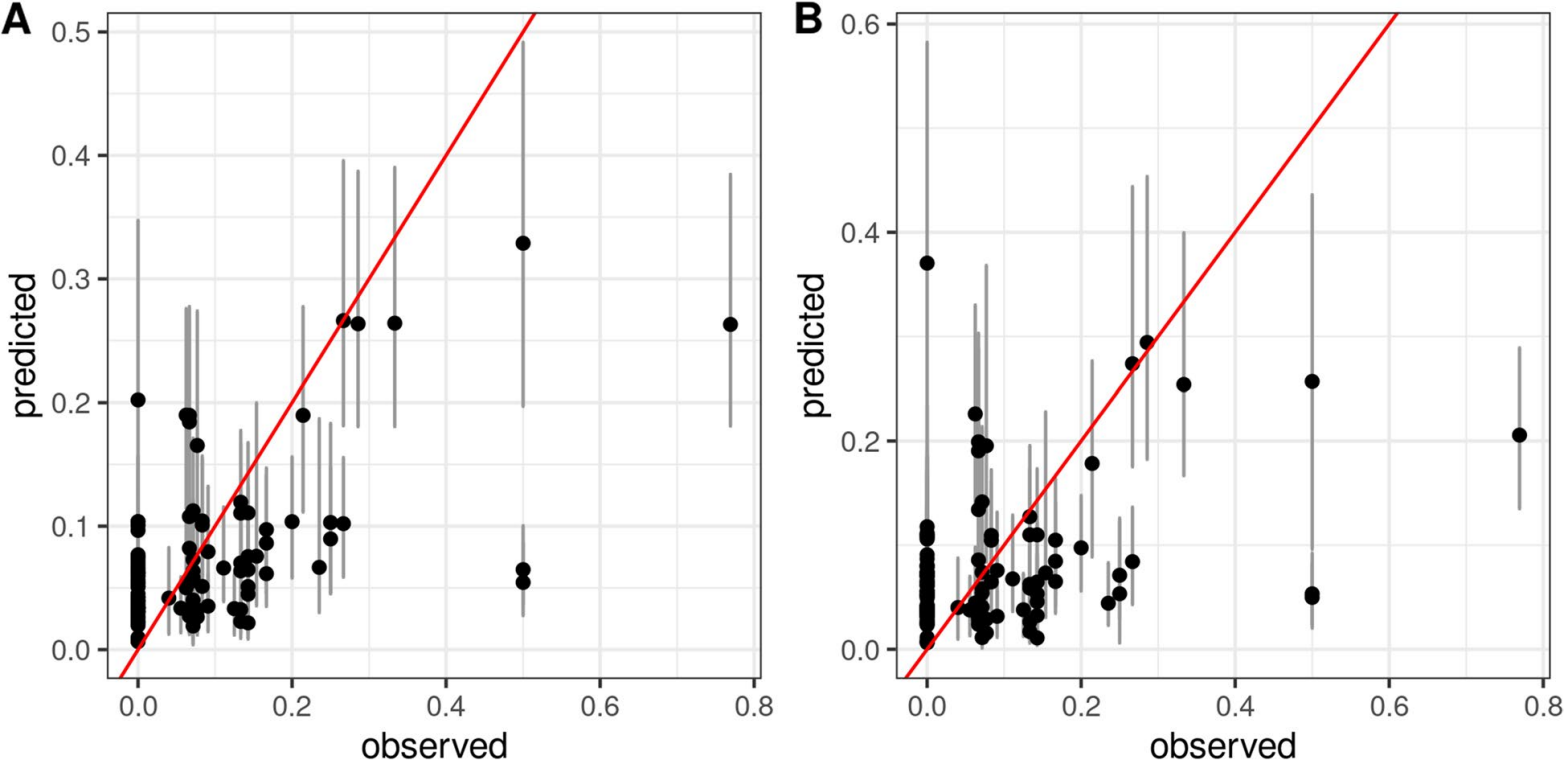
Observed levels of severe acute malnutrition (SAM) versus model predictions in Papua, Indonesia. The two plots show the results of in-sample comparisons for the final models (Panel A, left) and random, 10-fold cross-validation (Panel B, right). Diagonal lines are 1:1 lines where predictions equal observations

The selected covariates were used in the geostatistical model to predict the proportion of children under 2 years of age experiencing SAM at a 1 × 1 km spatial resolution across Papua. The posterior estimates of the model parameters (mean and 95% credible intervals) are reported in the supplemental materials (Supplementary Table 4, Additional File 1). Coefficients for covariates with 95% credible intervals that did not include zero were interpreted as having a consistent association with SAM. After controlling for the significant spatial effect, only precipitation showed a consistent positive association with the geographic variation in SAM. The predictive R^2^ was 0.33 and 0.15 for the in-sample and cross-validation comparison, respectively. The comparisons between observed and predicted values of SAM at the cluster level are shown in Fig. 2.

**Fig. 3:**
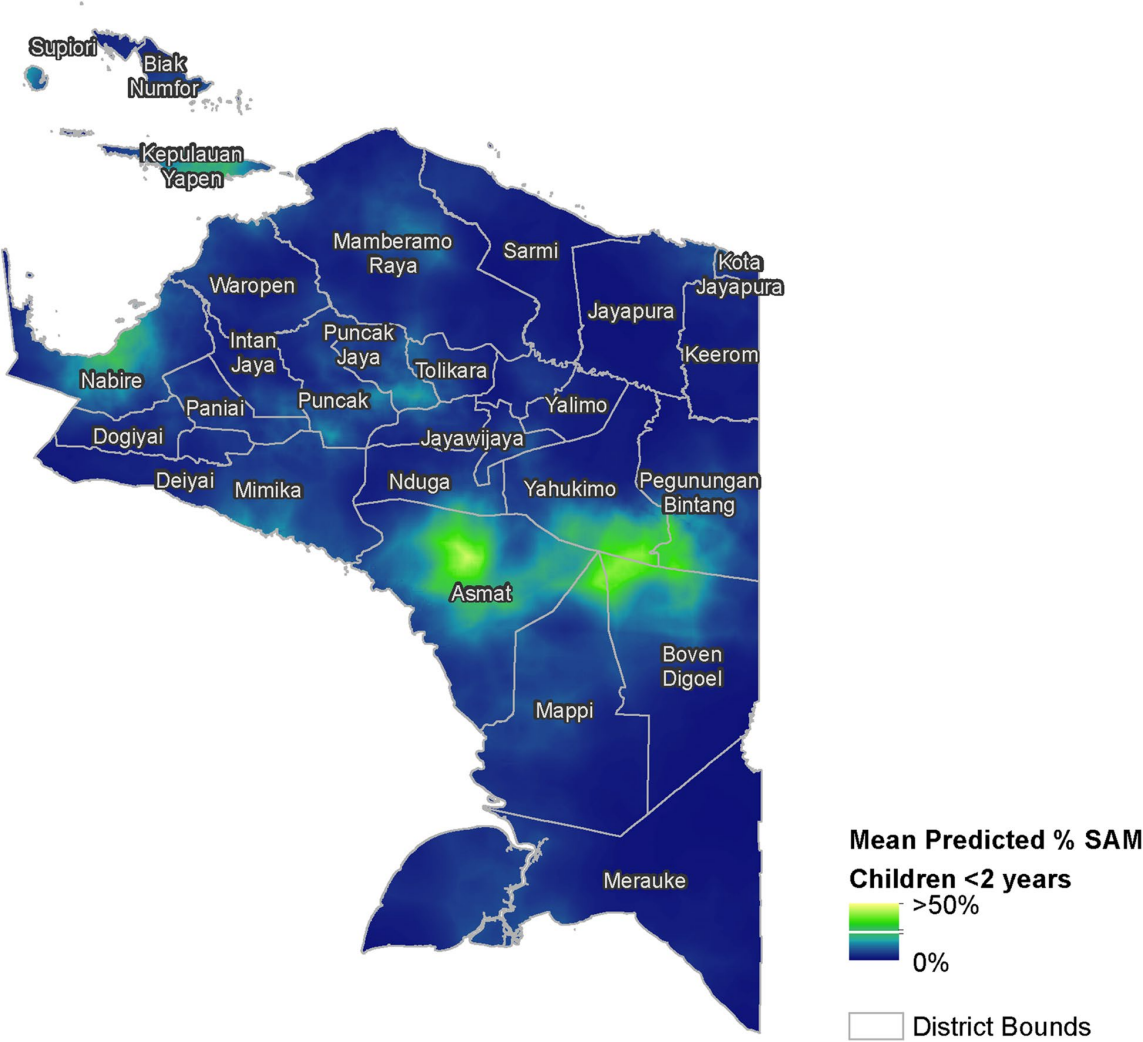
Mean predicted percentage of children < 2 years of age experiencing SAM in Papua, Indonesia. Predictions are based on model-based geostatistics and survey data from 2018. Data are shown at a 1 × 1 km spatial resolution

**Fig. 4:**
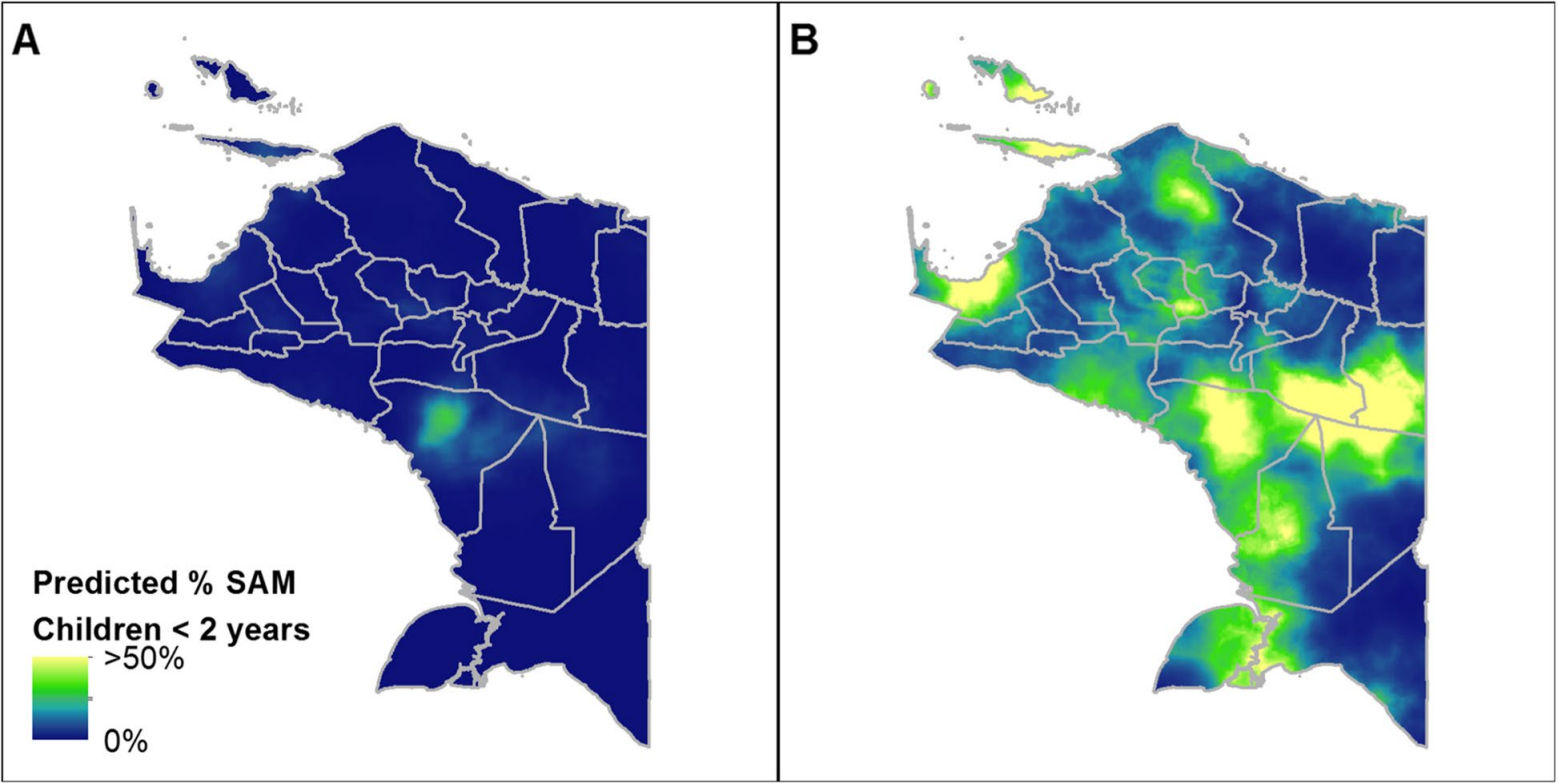
Lower (**A**) and upper (**B**) 95% prediction intervals for SAM in Papua, Indonesia. These predictions are based on the modelled estimates for 2018 survey data. Data shown are at a 1-km spatial resolution. Note that both maps are shown on the same color scale

The gridded output of the geostatistical model predicting the percentage of children under 2 years of age experiencing SAM is shown in Fig. 3. The lower and upper bounds of the predictions are shown in Fig. 4A and B, respectively, which highlights the spatial variation in the uncertainty of the predictions. In general, the highest levels of SAM were predicted in the district of Asmat and in the areas to the east of Papua. We note that the area in eastern Papua with high levels of SAM is in contrast to its nearest sample data locations (Fig. 1). This indicates that the high predicted levels of SAM in this area primarily reflect the combination of covariate values. The aggregated totals for each district are reported in the supplemental materials (Supplementary Table 1, Additional File 3). At the province level, the geostatistical model estimated that 6.3% (95% CI 4.2–10.9%) of Papuan children under 2 years of age were experiencing SAM in late 2018. This equates to approximately 15,213 children (95% CI 10,209–26,252).

**Fig. 5:**
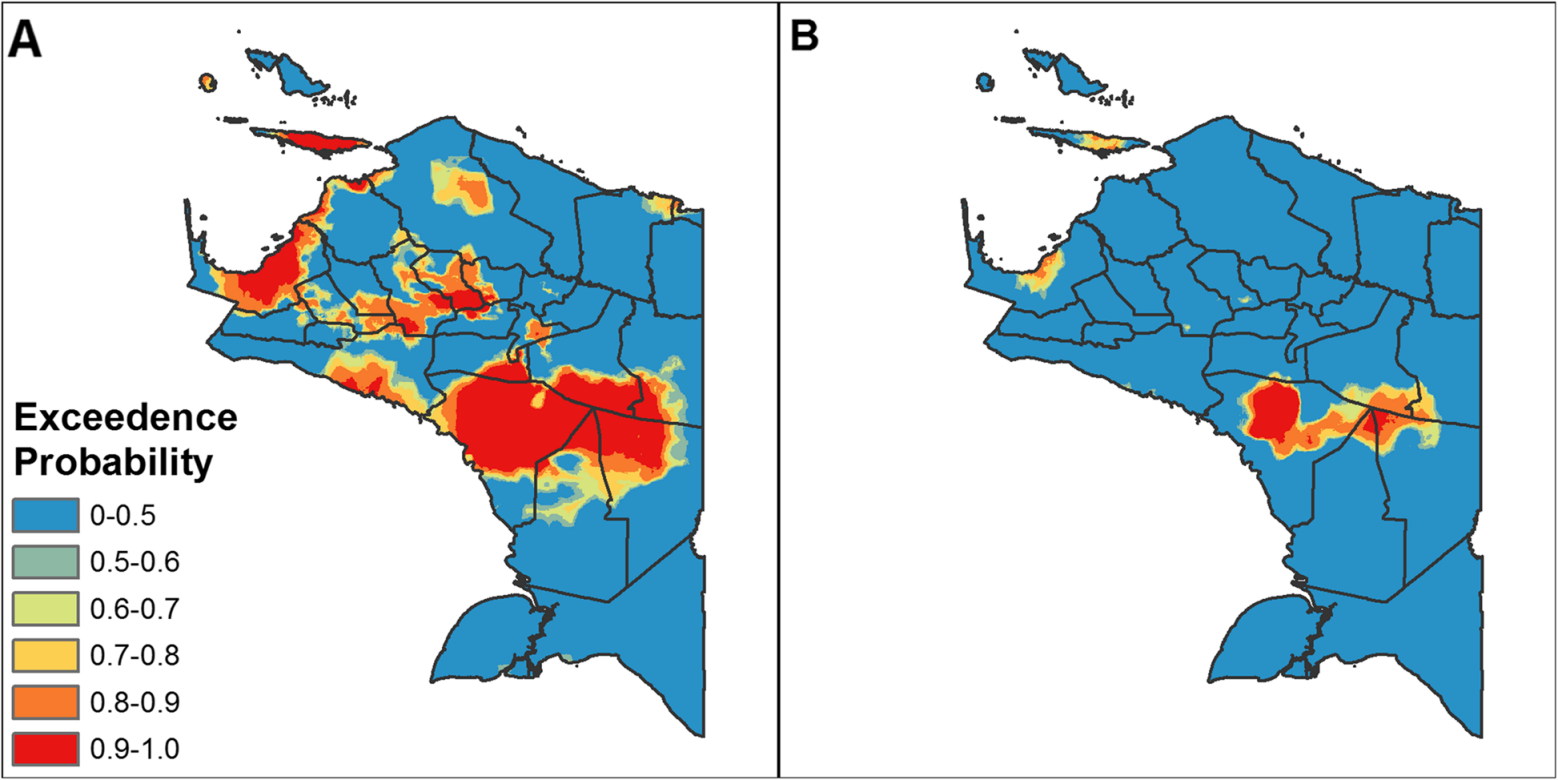
Probability that SAM exceeds the 5% (**A**) and 15% (**B**) prevalence thresholds in children under 2 years of age. Estimates are made for each 1 × 1 km grid cell. Note that exceedance maps for 2 and 15% thresholds are shown in Additional File 2

The probability that the predicted levels of SAM exceeded the thresholds of 5 and 15% of children under 2 years of age in a given 1 × 1 km grid cell is shown in Fig. 5A and B. The results for the 2 and 10% thresholds are shown in Additional File 2 (Supplementary Fig. 1). In this alternative mapping approach, higher values (shown in red) indicate that the model is more confident that the given threshold was exceeded. Despite the uncertainty around the mean predictions of SAM, these maps again highlight that children in areas around Asmat likely experienced very high levels of SAM.

## Discussion

Our study is the first to use geospatial modelling to predict SAM prevalence at a 1-km spatial resolution from sparse survey data, and our work presents some critical findings. We estimate that approximately 6.3% (95% CI 4.2–10.9%) of all Papuan children under 2 years of age were experiencing SAM in late 2018. Based on the geostatistical analysis, there are areas within Papua that very likely experienced even higher levels of SAM. Importantly, we used a Bayesian framework to estimate our models which allowed for uncertainty in the predictions to be quantified. Producing estimates on a gridded surface also allowed the results to be easily visualized, providing the flexibility to aggregate the gridded estimates into any geographically defined unit, which might be useful from a policy or programmatic perspective. We demonstrated this step by aggregating the gridded predictions to the level of districts in Papua. The results of the geostatistical model predicted the proportion of Papuan children under 2 years of age experiencing SAM. By combining these estimates with gridded estimates of the population at risk, we were also able to predict the total number of children who experienced SAM. The use of proportions should be compared with predicted estimates (counts) as they may have different implications in terms of policy responses. For example, although the prevalence of SAM in Asmat district (14%) is higher than that in Mimika district (8%), the total number of children affected by SAM in Mimika (2007) was much higher than that in Asmat (568 children).

The use of exceedance probabilities to express uncertainty in predictions exceeding thresholds of SAM can be particularly useful from a policy-making standpoint. For instance, certain areas of the Papua province were likely to be in a critical situation, with well over 15% of Papuan children under the age of two being severely acutely malnourished. This has important implications for malnutrition programming in Papua to target those most in need. Our analyses highlight that significant advances in addressing malnutrition are required in the province if it is to meet the WHO Global Nutrition Target (GNT) to reduce wasting prevalence to less than 5% or the UN SDG to end all forms of hunger and malnutrition by 2030 [22].

### Limitations

With reference to this particular study, the analysis has some limitations related to its source datasets. First, the distribution of the sample locations is not ideal for geostatistical modelling methods. Geostatistical models draw strength from spatial distribution of the sample sites and the assumption that areas near to observed samples are more similar. However, in this case study, the primary sampling units are located in a small number of districts, leading to a low spread of observations across the study area. That leaves large parts of the study area to be predicted from distant data points, which may lead to higher uncertainty in the predictions and it limits our ability to validate the outputs in these areas. Moreover, sites in close proximity (< 1 km) to one another may be coded as experiencing the same or very similar geospatial covariate values (due to the spatial resolution of the covariates). Therefore, it is difficult for the covariates to explain the observed differences in SAM among these clusters. The pattern of cluster locations may have affected the parameter estimates for the covariates and result is more uncertainty in the gridded predictions. Finer resolution covariates could be explored to account for this, though the predictions become more computationally challenging.

In addition, the source data are not representative in the same way that a national survey, such as the DHS or National Nutrition Surveys, would be. The baseline survey data were sampled from households in Papua where the caregiver self-identified as being of indigenous Papuan ethnicity [8]. We used this sample to examine the geographic variation in SAM, therefore our predicted SAM risk is most representative of that population of children in Papua. In the absence of estimates of the indigenous Papuan population, we used total population estimates to approximate the population distribution. If children of different ethnic groups in Papua experience higher (or lower) rates of SAM, then our estimates of the absolute number of children who were SAM—which rely on an estimate of the total population—could be under- (or over-) estimated. Future studies are needed to understand the distribution of different population groups in Papua and their risk of malnutrition.

Additionally, treatment districts for the Child Grant (BANGGA Papua) were specifically targeted and selected from the poorest districts in the province. We did not explicitly model this characteristic of the sample, but these factors were taken into account by controlling for accessibility and local context so that predictions in un-sampled areas were as accurate as possible. With only one set of source data for the Papua analysis, however, validation options for modelling were also limited. Cross-validation was employed to evaluate out-of-sample precision.

It should also be noted that SAM is a relatively fast-moving indicator of malnutrition. In this regard, SAM or wasting reflects acute or short-term malnutrition, while stunting reflects chronic or long-term malnutrition [23] and while we can predict SAM at one point in time, the prevalence of this indicator might have changed soon after measurement. Cross-sectional surveys, as used in this study, may not fully capture the fast-moving and changing risk of SAM. More waves of data at shorter time intervals (e.g. multiple times per season) could help identify “hot spots” with a consistently higher risk of SAM.

### Policy relevance and benefits

The use of modelling methods to combine geospatial data with sparse geolocated survey data to predict health outcomes at high resolution or into unsampled areas offers many potential benefits in planning programs and monitoring progress toward government targets and the SDGs.

As noted earlier, many areas within Papua are very remote and cannot be accessed securely due to outbreaks of violent conflict, making ground-level data collection expensive or impossible [8]. Our findings suggest that this approach could provide real benefits in similar contexts where data collection is not possible or traditional surveys might experience gaps in coverage, such as remote areas, conflict-affected states, or areas with security concerns.

While the baseline data for Papua covered only six districts, these modelling techniques enabled us to predict SAM prevalence for the entire province – including districts that were not initially included in the baseline survey. Some of these districts also had high predicted levels of SAM, illustrating how this approach enabled us to identify SAM hot-spots that could be targeted with interventions that are known to work when tackling child undernutrition, such as for example the WHO-recommended approach of community management of acute malnutrition (CMAM) and ready-to-use therapeutic foods (RUTF) in community settings [24, 25].

## Conclusion

Eradication of malnutrition remains a key development goal, particularly in the context of LMICs. The application of geospatial mapping to SAM is relatively unexplored. This study shows that such a technique can be used to improve the monitoring and timely estimation of populations at risk of malnutrition in a context where geo-referenced survey or evaluation data related to a specific subject (malnutrition) are available, even when such data were not specifically collected for the purposes of mapping.

Despite the limitations, we show that sparse survey data can be used to derive relevant insights on the geographical distribution of SAM both for surveyed and non-surveyed areas. These could inform programming strategies and government responses to target areas where models show that SAM prevalence consistently exceeds a certain threshold with a specific degree of certainty.

In particular, this technique enabled us to generate insights about the prevalence of SAM in a context where data is scarce. This approach could therefore be particularly useful in a low-income country context where data collection is expensive, and where areas are inaccessible due to, for example, remoteness, political instability, and violent conflict.

## Supplementary Information

**Additional file 1.**

**Additional file 2.**

**Additional file 3.**

## Data Availability

The datasets used and/or analyzed during the current study are available from the corresponding author on reasonable request.

## Abbreviations

CG: Child grant
CI: Confidence interval
CGF: Child Growth Failure
CMAM: Community Management of Acute Malnutrition
DHS: Demographic and health surveys
GNTs: Global nutrition targets
INLA: Integrated nested Laplace approximation
LMICS: Lower- and middle-income countries
MAM: Moderate acute malnutrition
MICS: Multi-indicator cluster surveys
MUAC: Middle upper arm circumference
NNS: National nutrition surveys
PSU: Primary sampling unit
RUTF: Ready-to-Use Therapeutic Food
SAM: Severe acute malnutrition
SDGs: Sustainable development goals
SMART: Standardized Monitoring and Assessment of Relief and Transitions
UN: United Nations
UNICEF: United Nations International Children’s Emergency Fund
SD: Standard deviation
WHO: World Health Organization
WHZ: Weight-for-height z-score
