# Supplementary material for: Mapping the prevalence of severe acute malnutrition in Papua, Indonesia by using geostatistical models
Source: BMC Nutr. 2022 Feb 14;8:13. doi: 10.1186/s40795-022-00504-z (PMC8842923; doi:10.1186/s40795-022-00504-z)
Supplement: Supplementary file 1 — Additional file 1. [file 40795_2022_504_MOESM1_ESM.docx]

# **Additional File 1**

The geospatial covariates listed in [Supplementary Table 1](#_heading=h.32hioqz) were obtained for 2018 in Papua, Indonesia to match the survey period. Covariates such as slope are considered time-invariant and do not match the survey years. For data with higher temporal resolution (e.g. precipitation), the annual average was used. Data preparation included transforming and reprojecting the data to a common geographic coordinate system and converting the data to a consistent raster (gridded) format. The rasterized variables have a 30 arc-second (approximately 1 km × 1 km) spatial resolution. Processing was performed using ESRI ArcGIS 10.4 software. The covariate values were extracted at the survey cluster point locations.

**Supplementary Table 1: Summary of potential geospatial covariate layers and data sources**

| **Category** | **Covariate** | **Source** |
| --- | --- | --- |
| Climate and Environment | Precipitation | Harris, I., Osborn, T.J., Jones, P. et al. (2020) ‘Version 4 of the CRU TS monthly high-resolution gridded multivariate climate dataset’, *Scientific Data* Vol 7 (109), doi.org/10.1038/s41597-020-0453-3 |
|  | Aridity | Harris, I., Osborn, T.J., Jones, P. et al. (2020) ‘Version 4 of the CRU TS monthly high-resolution gridded multivariate climate dataset’, *Scientific Data* Vol 7 (109), doi.org/10.1038/s41597-020-0453-3 |
|  | Land surface temperature | Wan, Z., S. Hook and G. Hulley (2015) MOD11A2 MODIS/Terra Land Surface Temperature/Emissivity 8-Day L3 Global 1km SIN Grid V006, distributed by NASA EOSDIS Land Processes DAAC, doi.org/10.5067/MODIS/MOD11A2.006. |
|  | Evapotranspiration potential | Harris, I., Osborn, T.J., Jones, P. et al. (2020) ‘Version 4 of the CRU TS monthly high-resolution gridded multivariate climate dataset’ *Scientific Data* Vol 7 (109), doi.org/10.1038/s41597-020-0453-3. |
| Land Use/Land Cover | Distance to cultivated lands | WorldPop (www.worldpop.org - School of Geography and Environmental Science, University of Southampton; Department of Geography and Geosciences, University of Louisville; Departement de Geographie, Universite de Namur) and Center for International Earth Science Information Network (CIESIN) and Columbia University (2018) Global High Resolution Population Denominators Project - Funded by The Bill and Melinda Gates Foundation (OPP1134076). |
|  | Enhanced vegetation index (EVI) | Didan, K.. (2015) MOD13A3 MODIS/Terra vegetation Indices Monthly L3 Global 1km SIN Grid V006, distributed by NASA EOSDIS Land Processes DAAC, doi.org/10.5067/MODIS/MOD13A3.006. |
|  | Night-time lights | WorldPop (www.worldpop.org - School of Geography and Environmental Science, University of Southampton; Department of Geography and Geosciences, University of Louisville; Departement de Geographie, Universite de Namur) and Center for International Earth Science Information Network (CIESIN), Columbia University (2018) Global High Resolution Population Denominators Project - Funded by The Bill and Melinda Gates Foundation (OPP1134076). |
| Topography | Slope | WorldPop (www.worldpop.org - School of Geography and Environmental Science, University of Southampton; Department of Geography and Geosciences, University of Louisville; Departement de Geographie, Universite de Namur) and Center for International Earth Science Information Network (CIESIN), Columbia University (2018) Global High Resolution Population Denominators Project - Funded by The Bill and Melinda Gates Foundation (OPP1134076). |
| Remoteness | Distance to built settlements | WorldPop (www.worldpop.org - School of Geography and Environmental Science, University of Southampton; Department of Geography and Geosciences, University of Louisville; Departement de Geographie, Universite de Namur) and Center for International Earth Science Information Network (CIESIN) and Columbia University (2018) Global High Resolution Population Denominators Project - Funded by The Bill and Melinda Gates Foundation (OPP1134076). |
|  | Distance to major roads | WorldPop (www.worldpop.org - School of Geography and Environmental Science, University of Southampton; Department of Geography and Geosciences, University of Louisville; Departement de Geographie, Universite de Namur) and Center for International Earth Science Information Network (CIESIN), Columbia University (2018) Global High Resolution Population Denominators Project - Funded by The Bill and Melinda Gates Foundation (OPP1134076). |
|  | Distance to urban centers | Florczyk A.J., Corbane C., Ehrlich D., Freire S., Kemper T., Maffenini L., Melchiorri M., Pesaresi M., Politis P., Schiavina M., Sabo F. and Zanchetta L.. (2019) GHSL Data Package 2019, EUR 29788 EN, Publications Office of the European Union, Luxembourg, 2019, ISBN 978-92-76-13186-1, doi:10.2760/290498, JRC 117104. |
|  | Accessibility | WorldPop (www.worldpop.org - School of Geography and Environmental Science, University of Southampton; Department of Geography and Geosciences, University of Louisville; Departement de Geographie, Universite de Namur) and Center for International Earth Science Information Network (CIESIN) and Columbia University (2018) Global High Resolution Population Denominators Project - Funded by The Bill and Melinda Gates Foundation (OPP1134076). |
|  | Distance to conflicts | Clionadh, R., Linke, A., Hegre, H. and Karlsen, J.. (2010) ‘Introducing ACLED-Armed Conflict Location and Event Data’, Journal of Peace Research Vol 47(5), pp. 651- 660. |
| Health | Malaria risk | Weiss et al. (12) ‘Mapping the global prevalence, incidence, and mortality of Plasmodium falciparum, 2000–17: a spatial and temporal modelling study’, Lancet,  DOI: [10.1016/S0140-6736(19)31097-9](https://doi.org/10.1016/s0140-6736(19)31097-9). |
|  | Diarrhea risk | Institute for Health Metrics and Evaluation (IHME). (2020) ‘Global Under-5 Diarrhea Incidence, Prevalence, and Mortality Geospatial Estimates 2000-2019’, Institute for Health Metrics and Evaluation (IHME), Seattle, United States of America, doi.org/10.6069/GZ76-SE92 |

Covariates were selected for use in the geostatistical model by using a multi-step procedure as described in the main text. The results of the quantitative selection steps are summarized below. Variable selection was performed using non-spatial logistic regressions. The AIC values from non-spatial logistic regression models using each covariate individually are summarized in [Supplementary Table 2](#_heading=h.1hmsyys) along with the variable inflation factors (VIFs) from a saturated model.

**Supplementary Table 2: Summary of covariate selection calculation steps for Papua, Indonesia**

|  | **AIC** | **P-values** | **VIF** | **Selected** |
| --- | --- | --- | --- | --- |
| Precipitation | 340.20 | 0.000 | 14.22 | X |
| Aridity | 339.78 | 0.000 | -- |  |
| Land surface temperature | 355.83 | 0.501 | 10.73 |  |
| Distance to conflicts | 354.13 | 0.137 | 9.02 | X |
| Distance to built settlement | 346.88 | 0.001 | 2.68 | X |
| Distance to cultivated land | 354.45 | 0.155 | 1.52 |  |
| Distance to major roads | 345.67 | 0.001 | 4.03 | X |
| Malaria risk | 353.45 | 0.108 | 4.34 |  |
| Enhanced vegetation index (EVI) | 349.67 | 0.008 | 2.54 |  |
| Evapotranspiration | 350.05 | 0.019 | 6.11 |  |
| Distance to urban centers | 331.20 | 0.000 | 6.85 | X |
| Slope | 344.38 | 0.002 | 4.58 |  |
| Urban accessibility | 355.96 | 0.565 | 4.75 | X |
| Night-time lights | 353.57 | 0.130 | 2.79 |  |
| Diarrhea risk | 355.87 | 0.516 | 6.45 |  |

The ‘Selected’ column indicates the variables chosen in the final process.

Pearson’s correlation was used to compare each set of covariates. While most covariates showed low correlations, aridity and precipitation were again highly correlated (R = 0.96). We removed aridity from consideration as a covariate based on the AIC values and visual comparisons of the scatterplots and maps. A summary of the correlations is shown in the heatmap in [Supplementary Figure 1](#_heading=h.3o7alnk).

###


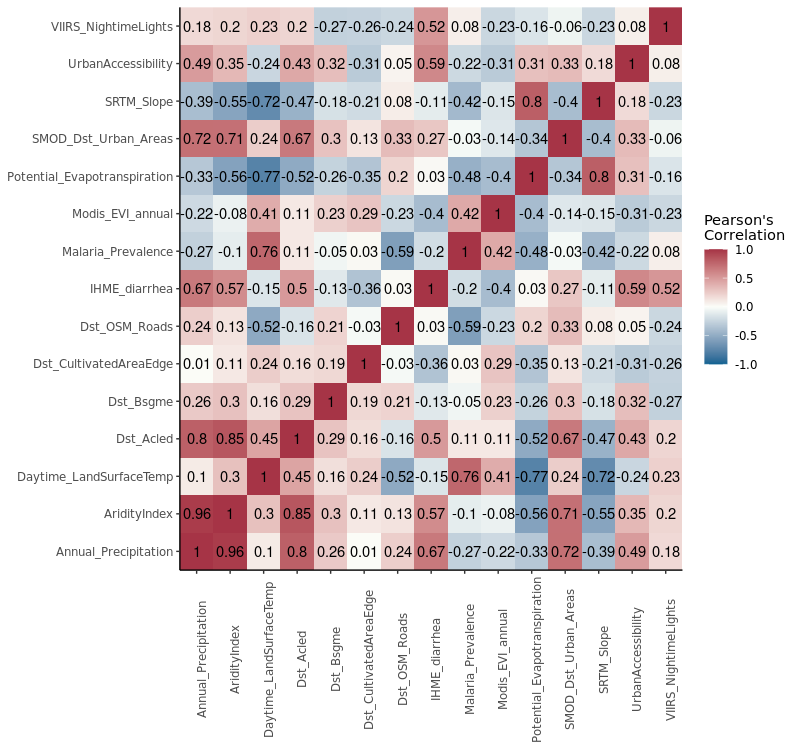


**Supplementary Figure 1: Correlation between pairs of covariate values at the survey cluster locations in Papua, Indonesia**

Finally, we applied a stepwise elimination based on AIC. The resulting non-spatial logistic regression model is shown in [Supplementary Table 3](https://docs.google.com/document/d/1aA0mlnbdtNuOnGKAVsplP5tBt1ZIVU49jkLACAk7WT8/edit#heading=h.nvziibjwx9t7). With this reduced set of covariates, the VIFs were each less than our threshold value of 5. The final selection of variables is also indicated in [Supplementary Table 3](https://docs.google.com/document/d/1aA0mlnbdtNuOnGKAVsplP5tBt1ZIVU49jkLACAk7WT8/edit#heading=h.nvziibjwx9t7).

**Supplementary Table 3: Final model based on stepwise-elimination variable selection for Papua, Indonesia**

|  | **Estimate** | **Std. Error** | **p-value** |
| --- | --- | --- | --- |
| (Intercept) | -2.8645 | 0.1253 | 0.000 |
| Precipitation | 1.0561 | 0.2476 | 0.000 |
| Distance to conflicts | -1.1675 | 0.2532 | 0.000 |
| Distance to built settlement | 0.3058 | 0.0982 | 0.002 |
| Distance to major roads | -0.3992 | 0.1413 | 0.005 |
| Distance to urban centers | 0.6417 | 0.1570 | 0.000 |
| Urban accessibility | -0.6120 | 0.1596 | 0.000 |

We used variogram modelling to examine the spatial variation of SAM among survey cluster locations. The observed levels of SAM exhibit some similarity across Papua, as seen in Supplementary Figure 2A with an effective range of approximately 101.0 km. Much of that similarity can be explained by geospatial data layers. A variogram of the residuals (Supplementary Figure 2B) of a model for SAM using the selected covariates (Supplementary Table 3) shows a much shorter effective range, approximately 19.6 km.

**Supplementary Figure 2: Variogram analysis of SAM at survey locations (A, left) and the variogram of residuals from a non-spatial model (B, right). The fitted variogram line use a Matern model.**


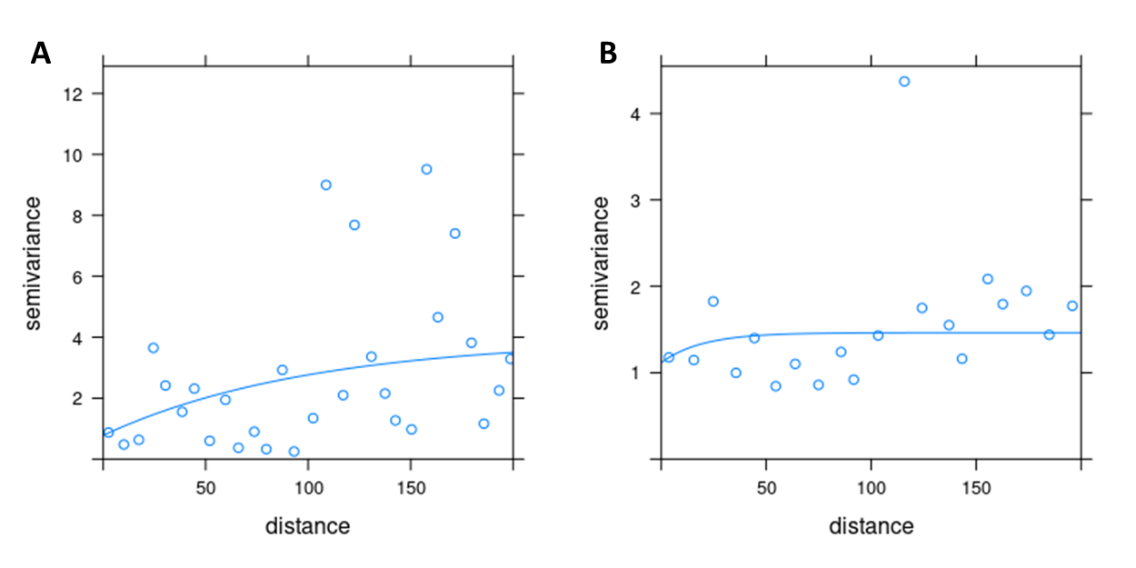


The selected covariates were used in a geostatistical model and estimated using Bayesian methods. The parameter estimates of the final model are reported in Table 4.

**Supplementary Table 4: Final geostatistical model for Papua, Indonesia**

|  | **Estimate** | **Std. Error** | **Lower 95%** | **Upper 95%** |
| --- | --- | --- | --- | --- |
| (Intercept) | -2.978 | 0.602 | -4.388 | -1.837 |
| Precipitation | 0.998 | 0.375 | 0.250 | 1.750 |
| Distance to conflicts | -1.032 | 0.44 | -1.778 | 0.001 |
| Distance to built settlement | 0.289 | 0.137 | -0.025 | 0.525 |
| Distance to major roads | -0.369 | 0.200 | -0.733 | 0.064 |
| Distance to urban centers | 0.461 | 0.450 | -0.757 | 1.044 |
| Urban accessibility | -0.511 | 0.288 | -1.041 | 0.122 |
| Range of Spatial Effect | 5.740 | 5.466 | 1.330 | 19.980 |
| Std. Dev. of Spatial Effect | 1.630 | 0.896 | 0.460 | 3.880 |
