# Supplementary material for: Mapping the prevalence of severe acute malnutrition in Papua, Indonesia by using geostatistical models
Source: BMC Nutr. 2022 Feb 14;8:13. doi: 10.1186/s40795-022-00504-z (PMC8842923; doi:10.1186/s40795-022-00504-z)
Supplement: Supplementary file 2 — Additional file 2. [file 40795_2022_504_MOESM2_ESM.docx]

# **Additional File 2**

The geostatistical model form follows a typical prevalence survey model, as discussed by Diggle and Giorgi (2016) and Giorgi et al. (2021). It consists of modelling the number of children experiencing SAM ($Y_{i}$) from the sample of children ($N_{i}$) in each cluster ($i$). These clusters are sampled at locations $s_{i}:i=1, \ldots, n$ over the region of Papua. We let $p(s)$ denote the latent probability of SAM for a child at location which leads to a binomial sampling model:

$$Y_{i}\sim Binomial\left( N_{i},p\left( s_{i} \right) \right)$$

$$logit\left( p\left( s_{i} \right) \right)={\text{x}\left( s_{i} \right)}^{\boldsymbol{⊺}}\beta\boldsymbol{+}z\left( s_{i} \right)$$

We use a logit-transform to model $p\left( s_{i} \right)$ as a combination of a matrix of covariates ($\text{x}(s_{i})$) with estimated parameters $\beta$, and a spatial effect ($z(s_{i})$). The covariates are observed at all survey locations as well as continuously across the region of interest, allowing for predictions in unsampled locations. The spatial effect is a zero-mean Gaussian Process assumed to be stationary and isotropic with covariance matrix sigma. In this work we use a Matern covariance structure. The model was estimated in a Bayesian framework using integrated nested Laplace approximations (INLA) for latent Gaussian models [(14)](https://www.zotero.org/google-docs/?4R2Tee). The stochastic partial differential equations (SPDE) approach [(15)](https://www.zotero.org/google-docs/?AP93IJ) was used for the spatial random effect to approximate a continuous spatial field.

To generate the aggregated estimated of SAM for districts and the province, we use the 1 km x 1 km gridded predictions from the geostatistical model. Each location in the grid is associated with 1000 posterior samples from the fitted model. Following Giorgi et al. (2021), a regional prevalence ($p(R_{k}$)) of an outcome can be estimated as:

$$p\left( R_{k} \right)=\frac{\int_{R_{k}} w\left( s \right)p\left( s \right)dx}{\int_{R_{k}} w\left( s \right)dx}$$

where $p(s)$ is a prediction and $w(s)$ is the population density at location $s$. A summation of the predictions over a grid is used to approximate the integrals for a given region ($k$), essentially a population-weighted mean. We apply this summary approach to the full set of 1000 posterior samples at each grid cell to generate district-level distributions of the predicted SAM. From these district-level distributions we report the mean and credible intervals.

Diggle Peter J and Giorgi Emanuele (2016). “Model-Based Geostatistics for Prevalence Mapping in Low-Resource Settings.” *Journal of the American Statistical Association*, 111:515, 1096-1120, DOI: [10.1080/01621459.2015.1123158](https://doi.org/10.1080/01621459.2015.1123158)

Giorgi Emanuele, Fronterrè Claudio, Macharia Peter M., Alegana Victor A., Snow Robert W. and Diggle Peter J. (2021). “Model building and assessment of the impact of covariates for disease prevalence mapping in low-resource settings: To explain and to predict.” *J. R. Soc. Interface*. 182021010420210104. <http://doi.org/10.1098/rsif.2021.0104>.
