# Supplementary material for: Mapping the prevalence of severe acute malnutrition in Papua, Indonesia by using geostatistical models
Source: BMC Nutr. 2022 Feb 14;8:13. doi: 10.1186/s40795-022-00504-z (PMC8842923; doi:10.1186/s40795-022-00504-z)
Supplement: Supplementary file 3 — Additional file 3. [file 40795_2022_504_MOESM3_ESM.docx]

# **Additional File 3 Supplementary Table 1: Estimated prevalence of severe acute malnutrition (SAM) in children < 2 years of age**

|  | **Predicted Prevalence of SAM** | | |  | **Predicted Rate of SAM** | | |
| --- | --- | --- | --- | --- | --- | --- | --- |
|  | **Mean Count** |  | **(95% Conf. Int.)** |  | **Mean (%)** |  | **(95% Conf. Int.)** |
| **Asmat** | 544.2 |  | (364.6, 873.3) |  | 13.8 |  | (0.09, 0.22) |
| **Biak Numfor** | 476.5 |  | (46.5, 2541.2) |  | 7.1 |  | (0.01, 0.38) |
| **Boven Digoel** | 248.3 |  | (108.8, 475.1) |  | 5.8 |  | (0.03, 0.11) |
| **Deiyai** | 346.3 |  | (131.1, 633.5) |  | 4.7 |  | (0.02, 0.09) |
| **Dogiyai** | 289.3 |  | (86.9, 859.1) |  | 4.5 |  | (0.01, 0.13) |
| **Intan Jaya** | 233.7 |  | (85, 498.4) |  | 5.4 |  | (0.02, 0.12) |
| **Jayapura** | 429.5 |  | (134.8, 906.2) |  | 4.5 |  | (0.01, 0.1) |
| **Jayawijaya** | 311.5 |  | (179.6, 684.6) |  | 5.3 |  | (0.03, 0.12) |
| **Keerom** | 75.4 |  | (34.9, 121.6) |  | 2.7 |  | (0.01, 0.04) |
| **Kepulauan Yapen** | 870.2 |  | (277.4, 2354.8) |  | 16.4 |  | (0.05, 0.44) |
| **Kota Jayapura** | 970.2 |  | (182.4, 1811.5) |  | 6.8 |  | (0.01, 0.13) |
| **Lanny Jaya** | 471.0 |  | (266.8, 971.6) |  | 4.3 |  | (0.02, 0.09) |
| **Mamberamo Raya** | 81.7 |  | (28.4, 164.4) |  | 3.4 |  | (0.01, 0.07) |
| **Mamberamo Tengah** | 502.3 |  | (138.8, 1648.9) |  | 4.2 |  | (0.01, 0.14) |
| **Mappi** | 320.1 |  | (82.3, 855.6) |  | 4.5 |  | (0.01, 0.12) |
| **Merauke** | 439.1 |  | (47.5, 2149.8) |  | 3.0 |  | (0, 0.15) |
| **Mimika** | 2083.0 |  | (534.1, 5580.1) |  | 7.9 |  | (0.02, 0.21) |
| **Nabire** | 1171.3 |  | (213.3, 2524.8) |  | 13.1 |  | (0.02, 0.28) |
| **Nduga** | 459.7 |  | (207.1, 1233.6) |  | 5.8 |  | (0.03, 0.16) |
| **Paniai** | 942.2 |  | (532, 1430.5) |  | 6.2 |  | (0.04, 0.09) |
| **Pegunungan Bintang** | 397.0 |  | (64.2, 1622.6) |  | 6.2 |  | (0.01, 0.25) |
| **Puncak** | 712.9 |  | (165.5, 1614.2) |  | 7.0 |  | (0.02, 0.16) |
| **Puncak Jaya** | 722.0 |  | (139, 1446.7) |  | 8.4 |  | (0.02, 0.17) |
| **Sarmi** | 51.8 |  | (11.4, 220.1) |  | 2.4 |  | (0.01, 0.1) |
| **Supiori** | 27.1 |  | (1.2, 165.5) |  | 3.9 |  | (0, 0.24) |
| **Tolikara** | 411.2 |  | (180.4, 936.1) |  | 4.4 |  | (0.02, 0.1) |
| **Waropen** | 91.3 |  | (53.3, 194.9) |  | 4.6 |  | (0.03, 0.1) |
| **Yahukimo** | 1183.4 |  | (229.6, 3929.3) |  | 8.9 |  | (0.02, 0.3) |
| **Yalimo** | 350.5 |  | (84.9, 1327.3) |  | 2.9 |  | (0.01, 0.11) |

Predictions are based on aggregated model-based geostatistical predictions in the districts of Papua, Indonesia.


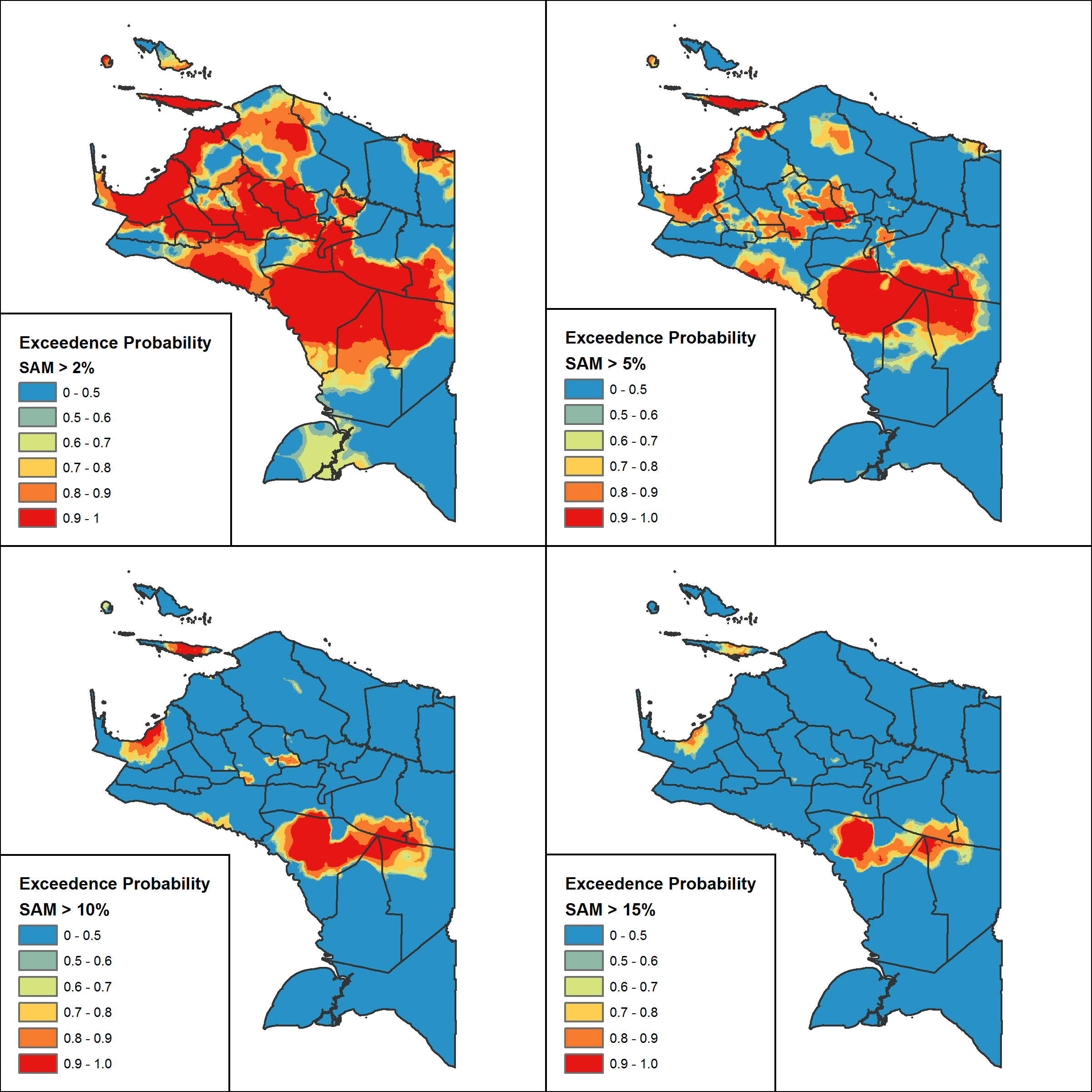


**Supplementary Figure 1: Probability of SAM exceeding the 2%, 5%, 10% and 15% prevalence thresholds in children under 2 years of age.** Estimates were made for each 1 × 1 km grid cell. Predictions were based on model-based geostatistical and survey data from 2018.
